# Isolation and Identification of Spoilage Fungi in Potato Fresh Wet Vermicelli and Inhibition Effect of Different Fungicides on Spoilage Fungi

**DOI:** 10.3390/jof11050367

**Published:** 2025-05-08

**Authors:** Feng Zhang, Mei Li, Jiachun Tian, Xia Ge, Shouqiang Li, Jianxin Chen, Yumei Li, Yaqian Zhang

**Affiliations:** 1 Agricultural Product Storage and Processing Research Institute, Gansu Academy of Agricultural Sciences, Lanzhou 730070, China; zhangfing0929@163.com (F.Z.);; 2 Gansu Innovation Center of Fruit and Vegetable Storage and Processing, Lanzhou 730070, China

**Keywords:** fresh wet vermicelli, spoilage fungi, isolation and identification, antimicrobial agents, antifungal effect

## Abstract

Fresh wet vermicelli is highly susceptible to microbial contamination during storage as a result of its high moisture content and rich nutrients, which leads to spoilage and deterioration. In addition to exerting a great impact on the quality of the product, this results in significant economic losses and potential food safety risks. This work aimed to identify spoilage microorganisms via traditional culturing methods and molecular biology techniques. The effects of environmental factors such as temperature and pH on the growth and development of the dominant spoilage fungi were investigated, and the inhibitory effects of both chemical (potassium sorbate) and natural antimicrobial agents (chitooligosaccharides, chitosan, tea polyphenols, citric acid, and ε-polylysine hydrochloride) were evaluated. The results indicated that *Penicillium crustosum* was the major spoilage microorganism in fresh wet vermicelli, whose optimal growth temperature and pH was 28 °C and 7, respectively. While conidial germination began at 7 h, hyphal formation was only observed after 12 h. Moreover, the findings suggest that both natural and chemical antimicrobial agents can effectively inhibit the growth of *P. crustosum,* with ε-polylysine hydrochloride being the strongest antimicrobial agent. Overall, the findings of this study provide a scientific foundation for improving the preservation of fresh wet vermicelli, which is of great significance for extending its shelf life and enhancing food safety.

## 1. Introduction

As a traditional Chinese food, fresh wet vermicelli is popular among consumers due to its smooth texture and rich nutritional value [1,2]. However, similar to fresh wet noodles, fresh wet vermicelli has a high moisture content and is rich in nutrients, making it highly prone to microbial contamination, which leads to spoilage and deterioration [3]. This not only affects product quality but also results in significant economic losses and food safety concerns [4]. In recent years, while scholars have conducted extensive research on spoilage microorganisms in food, studies specifically on spoilage microorganisms in fresh wet vermicelli remain limited. Jin et al. used high-throughput sequencing to identify *Bacillus aryabhattai* as the dominant spoilage bacterium in fresh starch noodles [5]. Bi et al. identified *Aspergillus* 305.01 and *Aspergillus* 305.02 as the spoilage fungi in wet starch noodles and investigated the effects of different ultra-high-pressure conditions on their inactivation [6]. Fungal contamination-induced mold growth is one of the primary causes of quality deterioration in fresh wet vermicelli. Contaminating fungi not only decompose nutrients in potato starch noodles, producing off-flavors and toxins, but also generate mycotoxins that pose severe threats to human health [7]. Under suitable temperature and humidity conditions, fungi can grow and reproduce rapidly, leading to the spoilage and deterioration of fresh wet vermicelli. As such, isolating and identifying spoilage fungi in fresh wet vermicelli, as well as clarifying their species and growth characteristics, is crucial for controlling fungal contamination and ensuring food safety.

Chemical preservatives have been widely used in the food industry in recent years due to their high efficacy. However, natural preservatives are gaining increasing attention given their safety and environmental benefits [8,9]. As chemical preservatives extend food shelf life by interfering with microbial growth and reproduction, concerns regarding their absolute safety remain controversial [10]. Natural preservatives, derived from plants, animals, and microorganisms, are primarily used in the food industry to inhibit undesirable microbial growth, which plays a crucial role in ensuring food safety and extending shelf life [11,12]. Previous work has shown that biofilms prepared using chitosan (CS), bacterial cellulose (BC), and ε-polylysine (ε-PL) exhibit excellent antibacterial properties, which significantly delay the spoilage of tilapia [13]. Tea polyphenols and chitosan have inhibitory effects on both Gram-positive and Gram-negative bacteria, effectively improving food safety and shelf life [14]. Moro et al. evaluated the antifungal effects of three organic acids (acetic acid, sorbic acid, and propionic acid) on *P. roqueforti*, *P. paneum*, *A. pseudoglaucus*, *A. montevidensis*, and *Hyphopichia burtonii* isolated from spoiled baked goods [15]. They found that these five spoilage fungi revealed different sensitivities to organic acids, with the antifungal capacity of organic acid preservatives significantly increasing at lower pH levels. Additionally, Buatong et al. found that chitosan–gallic acid conjugates were highly effective in inhibiting fungal contamination in salted dried fish [16]. As such, identifying effective antifungal agents to prevent spoilage microorganism growth in food and extend product shelf life is crucial for the development of the food industry.

In this study, we aim to (a) use traditional culture methods and molecular biology techniques to isolate and identify spoilage fungi in fresh wet vermicelli and determine their species, (b) investigate the effects of environmental factors such as temperature and pH on the growth of major spoilage fungi in fresh wet vermicelli and analyze their growth mechanisms to provide a theoretical basis for developing effective control measures, and (c) compare the inhibitory effects of six preservatives on spoilage fungi in fresh wet vermicelli to explore new preservation strategies and methods, thereby ensuring product quality and promoting industrial development.

## 2. Materials and Methods

### 2.1. Materials

Fresh wet vermicelli was produced by the ‘Dingxi Wide Noodles’ industrial park. The prepared fresh wet vermicelli was weighed to 200 g and put into a food packaging bag (non-vacuum) for sealing. Then, it was stored at the Agricultural Product Storage and Processing Laboratory of the Gansu Academy of Agricultural Sciences (25 ± 2 °C, 60–75% relative humidity) to monitor spoilage and deterioration during storage.

Potato dextrose agar (PDA) and Bengal red agar (RBC agar) were prepared in-house. In total, 200 g of potato, 20 g of glucose, 18 g of agar, and 1000 mL of distilled water were used to prepare the PDA. The RBC agar was prepared by adding 5 g of peptone, 10 g of glucose, 1 g of potassium dihydrogen phosphate, 0.5 g of magnesium sulfate, 18 g of agar, 0.033 g of Bengal red, and 0.1 g of chloramphenicol in 1000 mL distilled water. All materials were purchased from Lanzhou Wike Bioengineering Co. (Lanzhou, China). Agarose gel recovery kits were purchased from Allsheng Biotechnology Co., Ltd. (No. EG101-01, Beijing, China).

### 2.2. Methodology

#### 2.2.1. Spoilage Fungi Isolation and Purification

According to the modified method published by Ao et al., 5 g of moldy fresh wet vermicelli was cut and placed into a centrifuge tube. A total of 45 mL of sterile water was added into the tube under a laminar flow hood [17]. The suspension was serially diluted to concentrations of 10^0^, 10^−2^, 10^−4^, and 10^−6^ after vortex mixing. Then, 200 μL of each diluted solution was spread onto RBC agar plates, which were subsequently incubated at 28 °C for three days. Single colonies were transferred to PDA medium for further isolation and purification. The isolation and purification were performed for at least three rounds to ensure pure strains.

#### 2.2.2. Morphology Analysis of Spoilage Fungi

Purified fungi were cultured at 28 °C for seven days. Colony morphology, color, and spore characteristics were observed to preliminarily identify the fungi by comparing them with known fungal characteristics.

#### 2.2.3. PCR Analysis of Spoilage Fungi

Fungal strains that were cultured for three days were used for genomic DNA extraction and stored at −20 °C. According to the method published by Zhang et al. [18], the collected fungal mycelium was ground. Lysate was added and shaken for 15 min, then phenol–chloroform mixture was added, mixed thoroughly, and centrifuged. The supernatant was taken, and 1 mL of ethanol was added, mixed, and centrifuged again to discard the supernatant. Ethanol solution was added again and centrifuged to retain the precipitate. The precipitate was dissolved in 50 μL of sterile water for PCR analysis.

ITS1 (5′-TCCGTAGGTGAACCTGCGG-3′) and ITS4 (5′-TCCTCCGCTTATTGATATGC-3′) primers were designed using Primer Premier 5.0 to amplify the internal transcribed spacer (ITS) region of fungal rDNA [19]. PCR products were analyzed via gel electrophoresis, purified, and sent to Shanghai Shenggong Biotechnology (Shanghai, China) for sequencing. Sequences with >99.5% similarity in the NCBI database were used for fungal identification. Phylogenetic trees were constructed using the neighbor-joining method to infer evolutionary history. Evolutionary distances were computed using the Maximum Composite Likelihood method and expressed as units of the number of base substitutions per site. Evolutionary analyses were conducted in MEGA7. Final fungal species were determined based on morphological and molecular identification results.

#### 2.2.4. Spoilage Potential Measurement

After 7 d of cultivation, sterile water containing 0.05% Tween-20 was used to prepare the spore suspension. A hemocytometer was used to adjust the concentration of conidia to 1 × 10^6^ spores/mL.

Freshly produced potato starch noodles were treated with sodium hypochlorite for 3 min, rinsed, and dried under ultraviolet light for 30 min. A 50 μL spore suspension was spread onto the noodles, with sterile water as a control. The dried coated starch noodles were placed in preservation bags and stored at 28 °C. The spoilage and deterioration of the fresh wet noodles were observed, and pathogenic fungi were isolated and purified again to determine whether the obtained fungi were spoilage-related.

#### 2.2.5. Growth Characteristics of Spoilage Fungi

According to the method published by Wang et al. [20], 2 μL of a 1 × 10^6^ spores/mL spore suspension was inoculated onto PDA medium with different pH levels. The inoculated PDA medium was incubated at 28 °C for 7 d. The growth of fungal colonies and the colony diameters were observed and measured.

In addition, 2 μL of a 1 × 10^6^ spores/mL spore suspension was inoculated onto PDA medium and incubated at 4, 20, 25, 28, 30, and 35 °C for 7 d. The growth of fungal colonies and the colony diameters were observed and measured.

To examine spore germination dynamics, 2 μL of a 1 × 10^6^ spores/mL spore suspension was inoculated onto PDA medium disks and incubated at 28 °C for 4, 6, 7, 8, 9, and 10 h. The disks were observed under a microscope.

In total, 200 μL of a 1 × 10^6^ spores/mL spore suspension was inoculated into PDB medium and shaken at 28 °C for 12 h to study the development of spores. The growth of conidia was examined under a microscope.

#### 2.2.6. Antifungal Effects of Different Inhibitors on Spoilage Fungi

The stock solution was prepared by dissolving a total of 4 g of chitosan oligosaccharide, 0.3 g of chitosan, 1 g of citric acid, 0.5 g of potassium sorbate, and ε-polylysine hydrochloride in 10 mL of sterile water, respectively. After filtration through a 0.22 μm membrane, the solutions were added to sterilized PDA medium. The final concentrations of chitosan oligosaccharide, chitosan, and citric acid were 0, 5, 10, 20, 35, and 50 mg/mL, 0, 0.5, 1, 1.5, 2, and 2.5 mg/mL, and 0, 2, 5, 10, 15, and 20 mg/mL, respectively. The final concentrations of potassium sorbate and ε-polylysine hydrochloride were 0, 1, 2, 5, 10, and 15 mg/mL. The stock solution of tea polyphenols was prepared by dissolving 0.8 g of tea polyphenols in 2 mL of sterile water, and the stock solution was diluted to final concentrations at 0, 20, 50, 100, 150, and 200 mg/mL. After filtration, 200 μL of each diluted solution was spread onto PDA plates. PDA medium without addition (at a concentration of 0 mg/mL) was used as a control for each treatment.

A 2 μL aliquot of a 1 × 10^6^ spores/mL spore suspension was inoculated onto each PDA plate, which was subsequently incubated at 28 °C for 7 d. The growth of fungal colonies and the colony diameters were observed and measured, respectively.

### 2.3. Statistical Analysis

All experiments were repeated three times. Data were recorded and analyzed using Microsoft Excel 2020. All results were calculated as mean ± standard deviation. Statistical significance was analyzed using Duncan’s multiple range test (*p* < 0.05) with SPSS Statistics 26. Graphs were plotted using Origin95.

## 3. Results

### 3.1. Spoilage Symptoms of Fresh Wet Vermicelli During Storage

**Figure 1:**
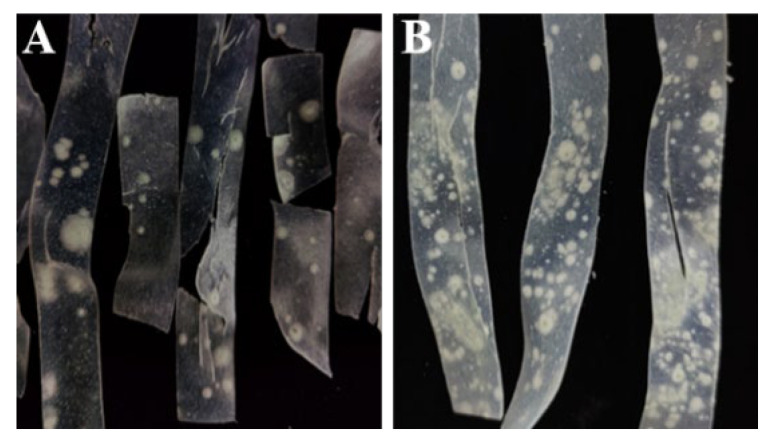
Symptoms of spoilage disease in fresh wet vermicelli when stored for 4 d (**A**) and 7 d (**B**).

The spoilage and deterioration of fresh wet vermicelli were monitored during storage. The results show that spoilage fungi began to appear on day 3, accompanied by a faint moldy odor. By day 5, noticeable fungal colonies in dot-like formations were observed on the surface of the vermicelli, with bluish-green spores developing in the center of the colonies (Figure 1A). By day 7, extensive fungal growth covered the vermicelli surface, with nearly 100% of the samples showing signs of spoilage and decay (Figure 1B). Before spoilage, we determined that the moisture content in fresh wet vermicelli is about 48–52%, which provided a suitable growth environment for microorganisms. As a result, potato fresh wet vermicelli is highly susceptible to microbial parasitism, especially *Penicillium*.

### 3.2. Morphology Observation of Spoilage Fungi

**Figure 2:**
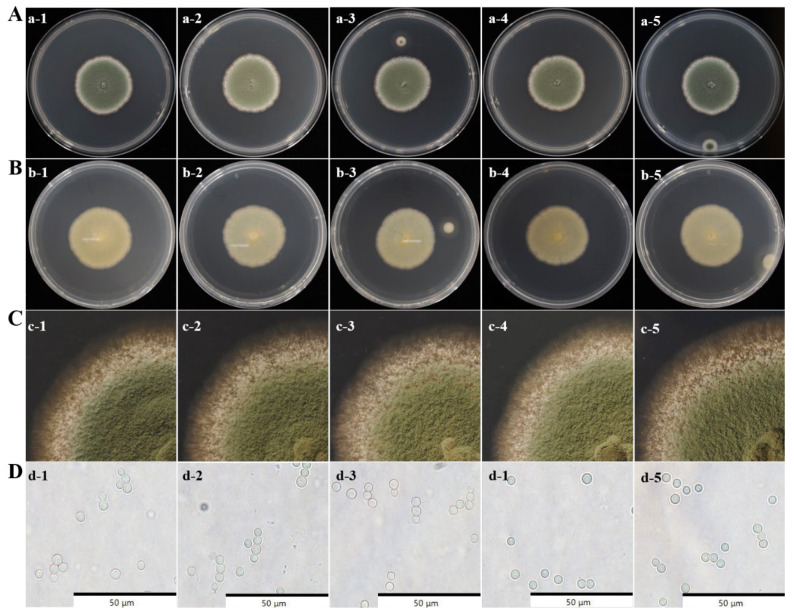
Frontal (**A**,**a-1**–**a-5**) and reverse (**B**,**b-1**–**b-5**) colony morphology, colony edge morphology (**C**,**c-1**–**c-5**), and spore size and geometry (**D**,**d-1**–**d-5**) of spoilage fungi.

In order to clarify the spoilage microorganisms of industrially produced potato fresh wet vermicelli, we picked vermicelli with obvious spoilage microorganism growth. Spoilage microorganisms were isolated and purified using traditional isolation methods, yielding five fungal strains with similar colony morphology. Observation of colony characteristics, colony edge hyphal growth, and spore size and shape revealed that these five strains formed relatively small colonies with a slightly raised center and bluish-green conidia on the surface (Figure 2A). The reverse side of the colonies appeared brown or milky white with a smooth texture (Figure 2B). The colony edges were neat and smooth, with dense, velvety hyphae (Figure 2C). The conidia were round or oval, with smooth walls, and had a diameter of approximately 2.5–3.5 μm. Based on morphological observations, these five strains were preliminarily identified as *Penicillium* species. This suggests that *Penicillium* is susceptible to growth on fresh wet vermicelli, providing support for our subsequent focus on what we want to preserve and preserve.

### 3.3. Molecular Analysis of Spoilage Fungi

**Figure 3:**
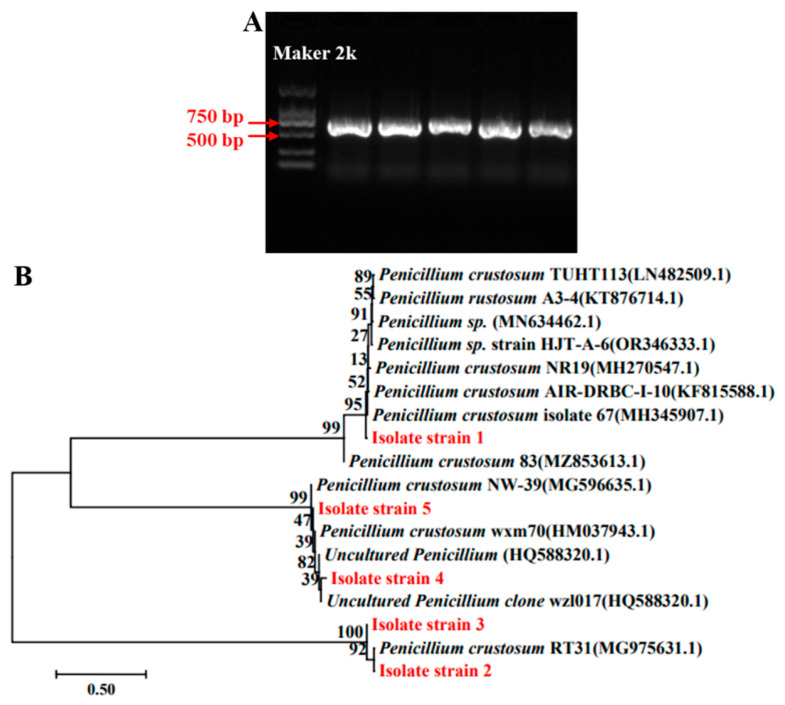
PCR amplification (**A**) and phylogenetic tree (**B**) of spoilage fungi. The strains isolated in this study are shown in red. Phylogenetic trees were constructed using the neighbor-joining method to infer evolutionary history. The optimal tree with a sum of branch length = 5.48738390 is shown. The percentage of replicate trees in which the associated taxa clustered together in the bootstrap test (500 replicates) are shown above the branches. The tree is drawn to scale, with branch lengths in the same units as those of the evolutionary distances used to infer the phylogenetic tree. The evolutionary distances were computed using the Maximum Composite Likelihood method and are in units of the number of base substitutions per site. The analysis involved 18 nucleotide sequences. Codon positions included were 1st + 2nd + 3rd + Noncoding. All positions containing gaps and missing data were eliminated. There was a total of 531 positions in the final dataset.

To further clarify the species identity of the isolated strains, the conserved ITS (internal transcribed spacer) sequence of fungi was analyzed. PCR amplification was conducted using the universal primers ITS1 and ITS4, resulting in bands of approximately 500 bp in size (Figure 3A). The amplified products were sequenced, yielding ITS sequences of about 550 bp. A BLAST comparison of the obtained sequences showed that all five isolated *Penicillium* strains shared more than 99.5% homology with *Penicillium crustosum*. The phylogenetic analysis revealed that isolate strain 1 was closely related to *P. crustosum* 67, while isolate strains 2 and 3 were closely related to *P. crustosum* RT31. Similarly, isolate strains 4 and 5 showed a close evolutionary relationship with *P. crustosum* wxm70 (Figure 3B). Based on morphological observation and molecular identification, we determined that the spoilage fungus was *P. crustosum*.

### 3.4. Spoilage Ability of the Fungal Isolates

**Figure 4:**
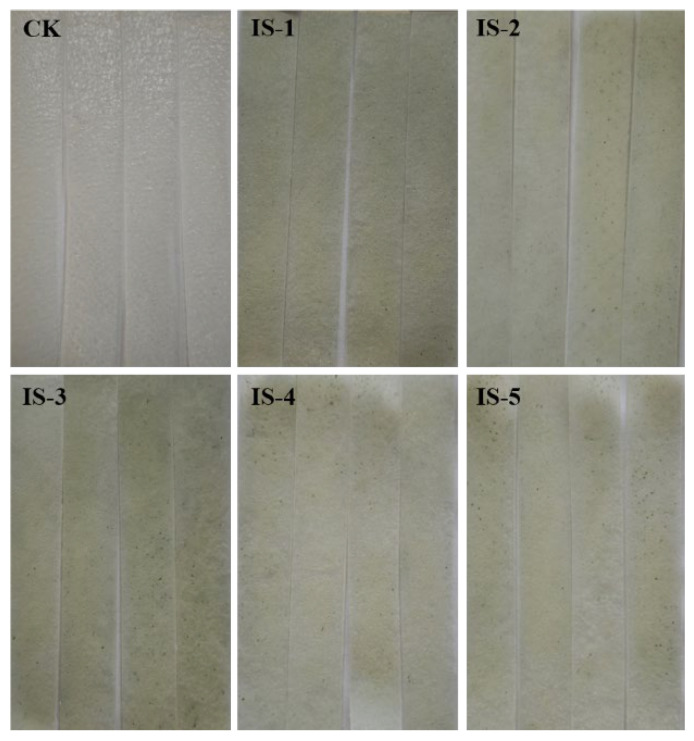
Spoilage and deterioration of spoilage fungi coated on fresh wet vermicelli at 5 d.

The five isolated *Penicillium* strains were inoculated onto fresh wet vermicelli to evaluate their spoilage capability and determine the dominant spoilage strain. After 5 d of incubation, all five *Penicillium* strains adhered to the surface of the vermicelli, with visible conidiospore formation. *Penicillium* isolate 3 exhibited the strongest spoilage ability compared to the other strains (Figure 4). The spoilage fungi were re-isolated and morphologically compared with the previously identified strains, confirming that the re-isolated fungi shared identical morphological characteristics. These findings indicate that the spoilage fungus responsible for the deterioration of fresh wet vermicelli is *P. crustosum*.

Since *P. crustosum*-3 demonstrated the highest spoilage potential among the isolates, it was selected for further investigation in subsequent studies.

### 3.5. Growth Characteristics of P. crustosum

#### 3.5.1. Colony Growth of *P. crustosum* at Different Temperatures and pH

**Figure 5:**
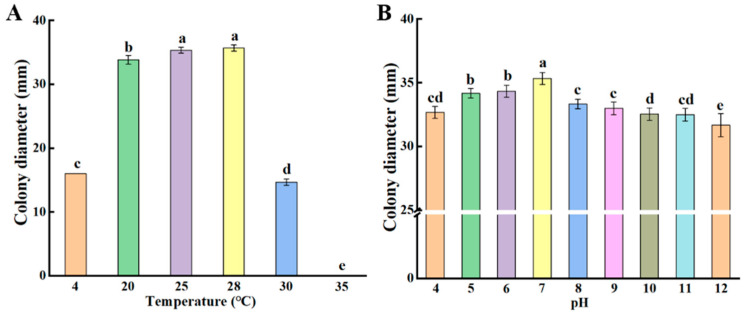
Colony growth at different temperatures (**A**) and pH (**B**) of *P. crustosum*. Bars indicate standard errors. Different letters indicate significant differences (*p* < 0.05).

The growth of *P. crustosum* was analyzed under various temperature conditions. The optimal growth temperature for *P. crustosum* was 28 °C, and the growth of *P. crustosum* was inhibited at 4 °C and 30 °C. *P. crustosum* was unable to grow at 35 °C (Figure 5A). In addition, the optimal growth pH of *P. crustosum* was studied; the neutral pH conditions were the optimal conditions for the fungus to grow, whereas acidic or alkaline conditions were unfavorable for its growth (Figure 5B). These findings suggest that the optimal growth conditions for *P. crustosum* are at 28 °C and pH 7. Clarifying its optimal growth conditions provides guidance for the subsequent process that we can use to reduce the contamination of *Penicillium* by adjusting the production and processing of fresh wet vermicelli.

#### 3.5.2. Kinetics of Conidial Germination of *P. crustosum*

**Figure 6:**
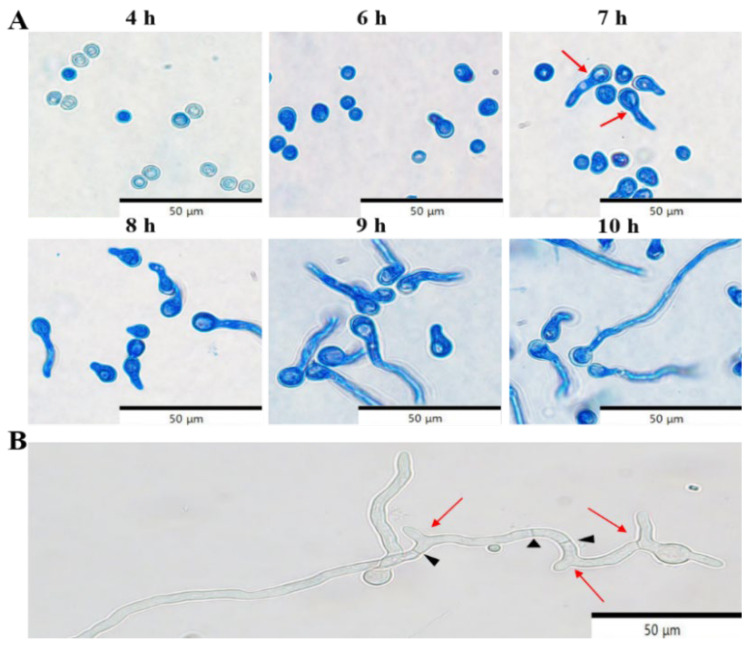
Conidial germination dynamics and stages of mycelial development of *P. crustosum*. Black arrows point to mycelial septa, red arrows point to bud tube bifurcation formation.

The kinetics of *P. crustosum* conidial germination were investigated. Initial signs of germination appeared at 6 h, and a small number of conidia had started to germinate by 7 h. Note that conidia were considered germinated when the germ tube length was more than twice the diameter of the conidium. Most conidia had germinated by 9 h, and germination was completed by 10 h (Figure 6A). After 12 h of shaking incubation, the developmental process of *P. crustosum* was examined with germ tube elongation, branching, and the formation of hyphal septa being observed (Figure 6B). These results indicate that *P. crustosum* conidia begin to germinate at 7 h, complete germination by 10 h, and develop hyphal branches and septa after 12 h. Observation of the growth and development of *Penicillium* can be prevented by effective control during its growth.

### 3.6. Inhibitory Effects of Different Antimicrobial Agents on P. crustosum

**Figure 7:**
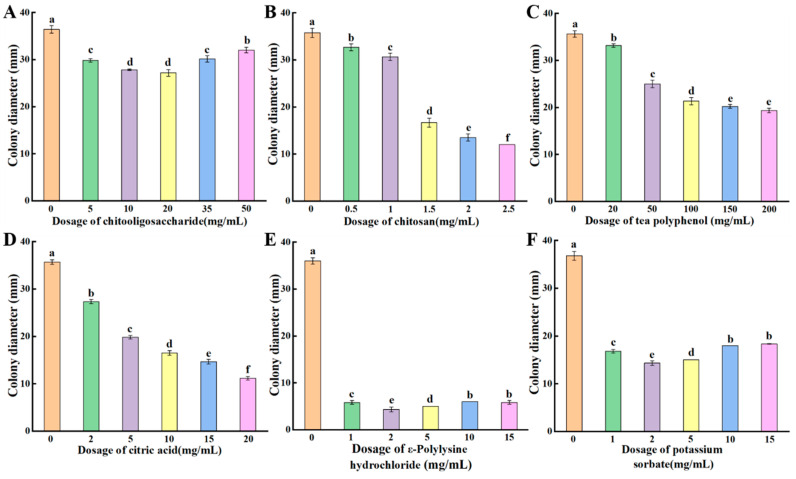
Inhibitory effect of chitooligosaccharide (**A**), chitosan (**B**), tea polyphenols (**C**), citric acid (**D**), potassium sorbate (**E**) and ε-polylysine hydrochloride (**F**) on *P. crustosum*. Bars indicate standard errors. Different letters indicate significant differences (*p* < 0.05).

The inhibitory effects of natural antimicrobial agents, including chitooligosaccharide, chitosan, tea polyphenols, citric acid, and ε-polylysine hydrochloride, along with the chemical preservative potassium sorbate, on *P. crustosum* were evaluated. The results showed that the inhibitory effects of chitooligosaccharide and ε-polylysine hydrochloride on *P. crustosum* initially increased with concentration but decreased at higher concentrations. The strongest inhibition was observed at chitooligosaccharide concentrations of 10–20 mg/mL (Figure 7A) and at an ε-polylysine hydrochloride concentration of 2 mg/mL (Figure 7E). Chitosan, tea polyphenols, and citric acid exhibited a dose-dependent inhibitory effect, with *P. crustosum* growth being progressively inhibited as their concentrations increased (Figure 7B–D). The inhibitory effect of potassium sorbate also showed a concentration-dependent pattern, initially increasing with concentration but decreasing at higher concentrations. The slowest growth of *P. crustosum* was observed at a potassium sorbate concentration of 2 mg/mL (Figure 7F). These findings demonstrate that both the chemical preservative potassium sorbate and the natural antimicrobial agents chitooligosaccharide, chitosan, tea polyphenols, citric acid, and ε-polylysine hydrochloride effectively inhibit the growth of *P. crustosum*. By determining the inhibitory effects of these inhibitors on *P. crustosum* in vitro, the screening of optimal inhibitors provides a scientific basis for the preservation of fresh wet vermicelli.

## 4. Discussion

Due to its high moisture content and rich nutrients, fresh wet vermicelli is highly susceptible to microbial contamination during storage, resulting in spoilage and deterioration [2,3]. In this work, *P. crustosum* is confirmed as the fungal species responsible for the spoilage of fresh wet vermicelli via both morphological observation and molecular analysis (Figure 2 and Figure 3). Bi et al. identified *Aspergillus* as the dominant spoilage mold in wet vermicelli [6], which may be attributed to differences in region, raw materials, and production processes. *Penicillium* species are widely distributed in the environment, revealing strong adaptability and reproductive capacity. Conidia can readily attach to food surfaces, causing spoilage and deterioration [21,22]. Previous studies have found that *Penicillium* species are the primary cause of spoilage in baked bread, followed by *Aspergillus* species [23,24]. Similarly, *Penicillium*, particularly *P. crustosum*, are the predominant cause for fungal spoilage in commercially produced sponge cakes in Montevideo [25]. Currently, there have been no reports on the predominant spoilage fungi in fresh wet vermicelli in China. To the best of our knowledge, this work is the first to identify *P. crustosum* as the dominant spoilage fungus in fresh wet vermicelli, providing important theoretical support for controlling fungal contamination and ensuring food safety.

The growth of spoilage fungi in fresh wet vermicelli is influenced by various factors, including nutrient composition, temperature, and pH. Most spoilage fungi grow rapidly at temperatures between 20 and 35 °C, and low temperatures can inhibit their growth [26,27]. In addition, the pH of fresh wet vermicelli typically ranges from 5 to 6.5, which is suitable for the growth of most spoilage fungi. In this study, we found that the optimal growth temperature for *P. crustosum* was 28 °C with no growth observed at 35 °C, and the optimal growth occurred at a pH of approximately 7 (Figure 5). A deeper understanding of the growth characteristics and developmental mechanisms of *P. crustosum* not only sheds light on the proliferation of spoilage fungi during the storage of fresh wet vermicelli but also supports the development of effective control strategies and innovative, eco-friendly preservation technologies.

Mold-induced spoilage is a serious issue during the storage of fresh wet vermicelli, making the use of preservatives an effective strategy for reducing spoilage and ensuring food safety. Currently, preservatives used in food include chemical preservatives (e.g., potassium sorbate) [28] and natural preservatives (e.g., chitooligosaccharide, chitosan, citric acid) [8]. Potassium sorbate is widely used in the food industry to effectively control microbial growth and extend the shelf life of food products [29]. However, excessive use of chemical preservatives may pose potential health risks [10]. Chitosan and chitooligosaccharide are two major deacetylated derivatives of chitin that possess excellent physicochemical properties and additional biological activities, making them widely used as natural antimicrobial agents in the food industry [30]. Citric acid, a natural organic acid, can alter environmental pH to disrupt the optimal growth conditions of microorganisms, thereby ensuring food safety [31]. As important secondary metabolites in tea, tea polyphenols contain bioactive compounds that interact with other food nutrients, influencing their physicochemical properties and functional activities. They also act as natural antimicrobial agents to inhibit the growth of spoilage microorganisms in food [32]. ε-Polylysine hydrochloride, a homopolymer of ε-polylysine, is a natural water-soluble polypeptide with excellent safety, stability, and broad-spectrum antifungal activity [33]. In this study, we evaluated the effects of six antimicrobial agents on the growth of *P. crustosum* and found that the five natural preservatives, namely chitooligosaccharide, chitosan, tea polyphenols, citric acid, and ε-polylysine hydrochloride, all exhibited inhibitory effects, with ε-polylysine hydrochloride showing the most significant inhibition (Figure 7). The chemical preservative potassium sorbate also inhibited the growth of *P. crustosum*, with an effective inhibitory concentration of 2 mg/mL (Figure 7). Yu et al. [34] reported that the fungicidal effects of chitosan were attributable to its inhibition effects on Cochliobolus heterostrophus by damaging conidial germination and appressorium formation. Yan et al. [35] reported that citric acid at various concentrations effectively inhibited spoilage bacteria in Guilin rice noodles, and an optimal inhibitory concentration of 120 mg/mL was reported. In addition, Li et al. [36] demonstrated that ε-polylysine hydrochloride disrupted the cell membrane structure of Alternaria species, resulting in nucleic acid and soluble protein leakage, thereby effectively inhibiting hyphal growth. Demirok et al. [37] found that chitosan treatment inhibited fungal growth on the surface of fermented sausages while decreasing the number of Gram (+) peroxidase (+) cocci, Enterobacteriaceae, molds, and yeasts in the sausages. Li et al. [38] Chitosan/ε-polylysine combination treatment retarded the growth of mycelium of *Aspergillus niger* and *Fusarium oxysporum*, destroying cellular structure, resulting in higher conductivity values, increased protein solubilization, increased extravasation of nucleic acids, and ultimately lethality. These findings suggest that natural antimicrobial agents can effectively inhibit the growth of spoilage fungi and serve as partial substitutes for chemical preservatives. Their antimicrobial efficiency can be enhanced for potential application in fresh wet vermicelli to extend shelf life through formulation optimization and process improvements.

## 5. Conclusions

In this work, *P. crustosum* was confirmed as the primary spoilage fungus in fresh wet vermicelli via both morphology observation and molecular analysis. The optimal growth temperature and pH for *P. crustosum* were determined to be 28 °C and 7, respectively. The germination of *P. crustosum* conidia was completed within 10 h, and hyphal formation began at 12 h. In addition, natural preservatives, such as chitooligosaccharide, chitosan, tea polyphenols, citric acid, and ε-polylysine hydrochloride, all revealed inhibitory effects on the growth of *P. crustosum*. ε-polylysine hydrochloride was reported as having the most pronounced impact. The chemical preservative potassium sorbate also effectively inhibited the growth of *P. crustosum* at a concentration of 2 mg/mL. This study provides valuable support for the future development of antimicrobial agents and high-efficiency preservation strategies for fresh wet vermicelli by identifying the dominant spoilage fungus in fresh wet vermicelli, understanding its growth characteristics, and systematically analyzing the effects of preservatives.

## Data Availability

The original contributions presented in the study are included in the article, further inquiries can be directed to the corresponding author.
